# Synergistic Modulation of Sn-Based Perovskite Solar Cells with Crystallization and Interface Engineering

**DOI:** 10.3390/molecules29112557

**Published:** 2024-05-29

**Authors:** Yunzhao Sun, Yaoyao Song, Mengfan Liu, Huiyin Zhang

**Affiliations:** School of Instrument Science and Opto-Electronics Engineering, Beijing Information Science &Technology University, Beijing 100192, China; syz13031926233@126.com (Y.S.); syy130635@163.com (Y.S.); 2023020215@bistu.edu.cn (M.L.)

**Keywords:** Sn-based perovskite solar cells, crystallization engineering, interface engineering, Sn^2+^ oxidation, carrier extraction

## Abstract

A high-quality Sn-based perovskite absorption layer and effective carrier transport are the basis for high-performance Sn-based perovskite solar cells. The suppression of Sn^2+^ oxidation and rapid crystallization is the key to obtaining high-quality Sn-based perovskite film. And interface engineering is an effective strategy to enhance carrier extraction and transport. In this work, tin fluoride (SnF_2_) was introduced to the perovskite precursor solution, which can effectively modulate the crystallization and morphology of Sn-based perovskite layer. Furthermore, the hole-transporting layer of PEDOT:PSS was modified with CsI to enhance the hole extraction and transport. As a result, the fabricated inverted Sn-based perovskite solar cells demonstrated a power conversion efficiency of 7.53% with enhanced stability.

## 1. Introduction

The remarkable development of organic–inorganic hybrid perovskite solar cells (PSCs) makes them important candidates for the next-generation photovoltaic technology due to the excellent optoelectronic properties [1,2,3,4,5,6]. The certified maximum efficiency has increased from 3.8% to the current 26.1%, close to that of silicon solar cells. At present, the research of PSCs is mainly based on lead (Pb) halide perovskite materials that delivered the highest power conversion efficiency (PCE) [7,8,9,10]. However, Pb element is toxic to human beings and is harmful to the environment. Therefore, it is necessary to construct Pb-free perovskites by using alternative elements with similar electronic characteristics, such as germanium (Ge) [11], tin (Sn) [12], antimony (Sb) [13], and bismuth (Bi) [14]. Among these elements, Sn is the most ideal candidate material to replace lead. Sn has a similar electronic configuration and also has an ionic radius most similar to that of Pb [15]. Sn-based perovskites inherit most of the excellent photoelectric properties of Pb-based perovskite. Furthermore, Sn-based perovskites have a narrower band gap, much closer to the ideal bandgap (1.3 eV) according to the Shockley–Queisser (S-Q) limit. Thus, theoretically, Sn-based PSCs have the capability to outperform their Pb-based counterpart, demonstrating better development prospects. However, the PCE and stability of Sn-based PSCs lag far behind those of Pb-based PSCs. Sn^2+^ is easily oxidized to Sn^4+^, which induces enormous defects and leads to intrinsic p-type doping in Sn-based perovskites [16,17,18,19,20]. These defects cause serious non-radiative recombination and performance loss. Meanwhile, the poor quality and coverage of Sn-based perovskite films due to the fast crystallization rate is also a challenge for the fast development of Sn-based PSCs [15,21,22,23,24]. High-quality perovskite film is the first principle to achieve high-efficiency Sn-based PSCs. Precursor engineering, additive engineering, and component engineering have been mainly developed to improve the stability of Sn^2+^ and the crystallization of Sn-based perovskite films [25,26,27,28,29,30]. Solvent engineering can effectively protect Sn^2+^ in the precursor from oxidation and modulate the crystallization rate. Hao et al. first replaced N,N-dimethylformamide (DMF) with DMSO in perovskite precursor solution to prepare Sn-based perovskite films. The strong complexation of DMSO can retard the crystallization of Sn-based perovskite films and form the intermediate phase of SnI_2_·3DMSO [31]. This intermediate phase not only retards the crystallization rate but also can effectively inhibits the oxidation of Sn^2+^. Additive engineering is the most used strategy to inhibit the oxidation of Sn^2+^, such as tin halides (SnF_2_ and SnCl_2_) [32], reducing agents [33], and small-molecule Lewis bases [34]. Therefore, tin fluoride (SnF_2_) additive is the most widely adopted and is unavoidable in the optimized fabrication of Sn-based perovskite films. The addition of SnF_2_ can increase the formation energy of Sn vacancy and compensate for the formed vacancy in Sn-based perovskite films [35]. In 2014, Kumar et al. first attempted to improve the photoelectric performance of CsSnI3-based perovskite solar cells by adding different proportions of SnF_2_. They demonstrated theoretically that the formation energy of Sn vacancies (V_Sn_) in CsSnI_3_ gradually increased with the increase in SnF_2_ concentration [36] Hartmann et al. systematically studied the effect of SnF_2_ on the photoelectric performance of CsSnBr_3_ and proved that a certain proportion of SnF_2_ can inhibit the oxidation of Sn^2+^ [37]. Subsequently, Koh et al. added SnF_2_ into FASnI_3_. They found that, during the spin-coating process, SnF_2_ would be precipitated preferentially, creating more nucleation sites and making the perovskite film more compact [38]. Zillner et al. also investigated the impact of SnF_2_ additives on interface formation in all lead-free FASnI_3_ perovskite solar cells and found that SnF_2_ preferably accumulates at the poly(3,4-ethylenedioxythiophene)/poly(styrenesulfonate) (PEDOT:PSS)/perovskite interface [39]. Nowadays, SnF_2_ has been widely used as a default additive to prepare efficient tin-based perovskite solar cells.

At present, high-performance Sn-based PSCs are almost based on inverted device configurations. Compared with the traditionally forward PSCs, inverted PSCs have almost no hysteresis. In inverted Sn-based PSCs, PEDOT:PSS is usually adopted as the hole transport layer, which is a well-studied highly conductive polymer with high visible light transmittance, good solution processability, and excellent perovskite precursor wettability [40]. The property of PEDOT:PSS is of vital importance to the efficiency and durability of inverted Sn-based PSCs. Except for high-quality perovskite active layer, improved interface charge extraction and transport derived from PEDOT:PSS is also crucial to achieve high-performance Sn-based PSCs [41,42]. Thus, it is necessary to optimize the properties of PEDOT:PSS, such as hydrophilicity, work function, surface morphology, and electrical conductivity [43]. Yang et al. modified the PEDOT:PSS with nicotinamide, and the work function of the hole-transporting layer shifted from −5.2 to −5.5 eV, optimizing the energy level matching. At the same time, the utilization of nicotinamide can improve the hydrophobicity of PEDOT:PSS and reduce the interface defects between PEDOT:PSS and the perovskite layer. As a result, the VOC and PCE of the tin-based perovskite device based on nicotinamide-modified PEDOT:PSS was increased to 0.83 V and 8.28%, respectively, under AM 1.5 G illumination [5]. Guo et al. selected polyethylene glycol (PEG) as both the etchant and passivator to optimize the PEDOT:PSS hole-transporting layer. PEG can etch the excess bulk PEDOT:PSS in the conventional spin-coating process, forming an ultrathin PEDOT:PSS layer with PEG molecules and rich PEDOT domains remaining on its surface, which can tune the work function of the PEDOT:PSS for better energy level matching [44]. 

Herein, both crystallization and interface engineering were applied to synergistically improve the performance of Sn-based PSCs. Firstly, SnF_2_ additive was introduced to optimize the crystallization and suppress the oxidation of Sn^2+^ in the Sn-based perovskite film. The morphology and photoelectric properties of Sn-based perovskite film with SnF_2_ were obviously improved, which is primary to high-efficiency Sn-based PSCs. Subsequently, CsI was used to modify the PEDOT:PSS hole-transporting layer. With the modification of CsI, the PEDOT:PSS layer exhibited enhanced hole extraction and transport ability, which is crucial to the further improvement in device performance. Due to the synergistic modulation of crystallization and interface engineering, a power conversion efficiency of 7.53% was achieved for the Sn-based PSCs with enhanced stability.

## 2. Results

### 2.1. Crystallization Engineering

**Figure 1:**
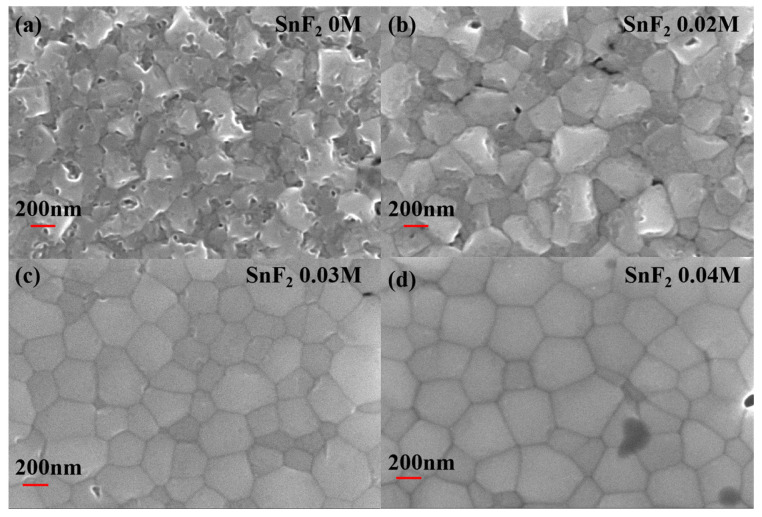
Top-view of SEM images of Sn-based perovskite films prepared with different SnF_2_ concentrations: (**a**) 0 M, (**b**) 0.02 M, (**c**) 0.03 M, and (**d**) 0.04 M.

The morphology and crystallization of perovskite films are crucial for the device performance. Here, SnF_2_ is introduced to improve the quality of Sn-based perovskite films. Different concentrations (0 M, 0.02 M, 0.03 M, and 0.04 M) of SnF_2_ in the perovskite precursor solution were compared to obtain the optimal concentration. Figure 1 shows the Scanning Electron Microscopy (SEM) images of Sn-based perovskite films prepared with different SnF_2_ concentrations. The introduction of SnF_2_ can obviously improve the morphology and crystallization of Sn-based perovskite films. The pristine perovskite film without SnF_2_ shows rough surface morphology and grain boundaries are not obvious (Figure 1a), which result in serious recombination. When 0.02 M SnF_2_ was added to the perovskite precursor solution, grain boundaries became clear, but the surface was still rough (Figure 1b). When the concentration of SnF_2_ increased to 0.03 M and 0.04 M, the obtained perovskite films were smooth and homogeneous. And crystal grains and grain boundaries can be easily distinguished in Figure 1c,d. Thus, it can be confirmed that SnF_2_ additive can effectively promote the crystallization and improve the morphology of Sn-based perovskite films. It has been reported that, due to the poor solubility in the perovskite precursor solution, SnF_2_ first precipitate homogeneously during the spin-coating process, functioning as heterogeneous nucleation sites to facilitate the growth of Sn-based perovskite crystals and enable a more uniform perovskite film [45]. Therefore, SnF_2_ is an effective additive in improving and maintaining the crystal quality of perovskite films for Sn-based PSCs.

**Figure 2:**
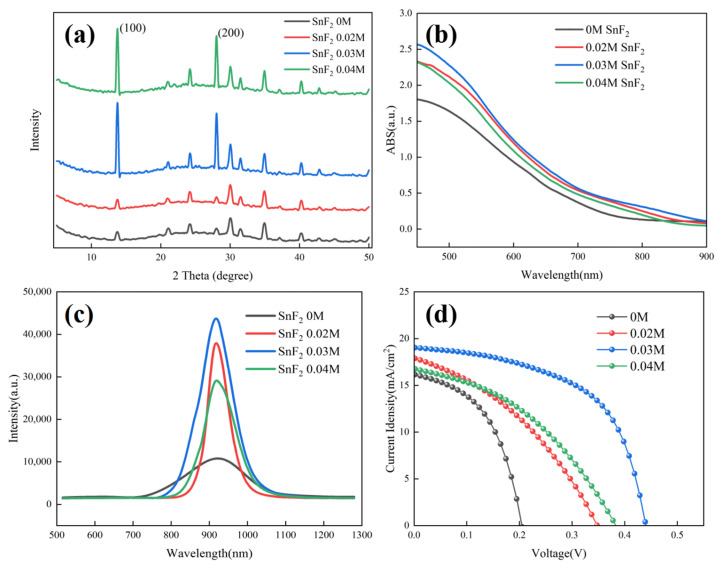
Characterization analysis of Sn-based perovskite films and PSCs prepared with different SnF_2_ concentrations: (**a**) XRD patterns of Sn-based perovskite films, (**b**) UV-vis absorption spectra of Sn-based perovskite films, (**c**) PL spectra of Sn-based perovskite films, and (**d**) J-V curves of Sn-based PSCs.

The effect of SnF_2_ on the crystallization of Sn-based perovskite films was further examined by X-ray diffraction (XRD). Figure 2a shows the XRD patterns of Sn-based perovskite films prepared with different SnF_2_ concentrations. It can be found that, when the concentration of SnF_2_ exceeded 0.03 M, the diffraction peaks of Sn-based perovskite films were obviously enhanced. Furthermore, 0.03 M SnF_2_ showed the highest diffraction peak, demonstrating the best crystallization. Moreover, all perovskite films showed (100) and (200) preferred growth with the diffraction peaks at 14.1° and 28.2°, respectively. Moreover, no diffraction peak position shift was found when SnF_2_ was added, indicating that SnF_2_ is not incorporated into the perovskite crystal structure, as is previously reported [39]. UV-vis absorption was invested to observe the spectral response range of perovskite films. Figure 2b shows the absorption profiles of different perovskite films. Compared to the pristine film, the absorption of perovskite films with SnF_2_ additive was obviously increased, indicating better crystallization consistent with the results of SEM and XRD. As the active absorbers, less non-radiative recombination, long carrier lifetime, and effective charge transport are demanded for high-performance devices. The photogenerated charge behavior of different Sn-based perovskite films were investigated using photoluminescence (PL) measurements. Figure 2c shows the PL spectra of different perovskite films deposited on insulating substrates, respectively. With the addition of SnF_2_, the PL intensity of perovskite films obviously improved, further identifying the crucial role of SnF_2_ in promoting crystallization and reducing defects of perovskite films. The reduced defects are beneficial in suppressing carrier recombination and facilitating carrier transport, delivering high device performance. The perovskite film with 0.03 M showed the highest PL intensity, implying the best device performance. The Sn-based PSCs, with an inverted configuration of ITO/PEDOT:PSS/FA_0.75_MA_0.25_SnI_3_/C60/BCP/Ag, were fabricated based on perovskite films with and without SnF_2_. Figure 2d presents the J-V curve to evaluate the photovoltaic performance of different devices. It can be identified that the addition of SnF_2_ can improve the device performance significantly. The device with 0.03 M SnF_2_ demonstrated the best photovoltaic performance consistent with the results of UV-vis and PL. The detailed photovoltaic parameters of Sn-based PSCs based on different SnF_2_ concentrations are listed in Table 1. The best device with 0.03 M demonstrated a PCE of 4.68%, with an open-circuit voltage (Voc) of 439.6 mV, a short-circuit current density (Jsc) of 19.04 mA/cm^2^, and a fill factor (FF) of 55.93%. However, the achieved PCE is unsatisfactory, and it is necessary to further optimize the device configuration.

### 2.2. Interface Engineering

**Figure 3:**
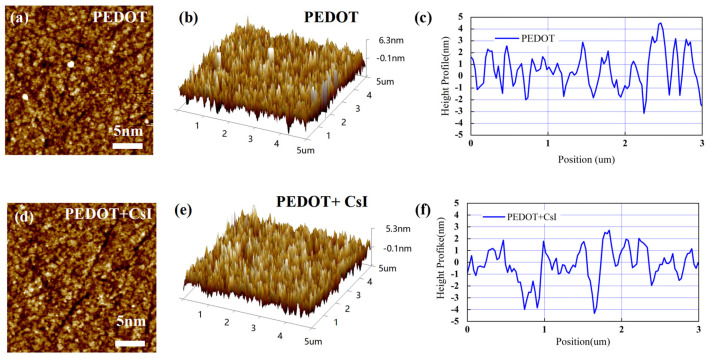
AFM morphology images and corresponding height profiles of PEDOT and PEDOT + CsI films: (**a**,**d**) two-dimensional topography, (**b**,**e**) three-dimensional topography, and (**c**,**f**) height profiles.

In this work, an inverted device configuration has been adopted to prepare the Sn-based PSCs with PEDOT:PSS as the hole-transporting layer. PEDOT:PSS is commonly adopted for high-performance Sn-based PSCs due to the suitable work function. However, PEDOT:PSS exhibits high recombination velocity at the interface with perovskite. Thus, it is desired to modify the PEDOT:PSS thin films, tailoring the interface toward an efficient and stable extraction of hole charge carriers. Here, we doped PEDOT:PSS with CsI to improve its photoelectric property and film morphology. As shown in Figure S1, when the concentration of CsI is 4 mg/mL, the Sn-based PSCs demonstrated the best device performance. In the following discussion, the effect of CsI (4 mg/mL) will be mainly compared with the control group. Firstly, atomic force microscopy (AFM) measurements were carried out to study the morphology change in the PEDOT:PSS film before and after adding CsI (4 mg/mL) (abbreviated as PEDOT film and PEDOT + CsI film). As shown in Figure 3a,d, the morphology of PEDOT + CsI film was improved. Agglomerated particles (bright sites) can be evidently found distributed on the surface of the PEDOT film, which is due to the agglomeration of PEDOT:PSS in the aqueous solution. Further, the surface roughness obtained from two-dimensional topography was evaluated to be 1.41 nm and 1.23 nm for the PEDOT film and the PEDOT + CsI film, respectively, denoting that CsI makes the PEDOT:PSS film smoother and more uniform. The results of three-dimensional topography (Figure 3b,e) were in accordance with that of two-dimensional topography. The height profiles across the grains were further obtained from the AFM morphologies, as shown in Figure 3c,f. All these results verified the smoother and more uniform surface morphology of the PEDOT + CsI film, which will provide a better interfacial contact that is beneficial for charge selection and recombination suppression. It can be concluded that the introduction of CsI can improve the dispersion of PEDOT:PSS in the aqueous solution and achieve a more uniform film.

**Figure 4:**
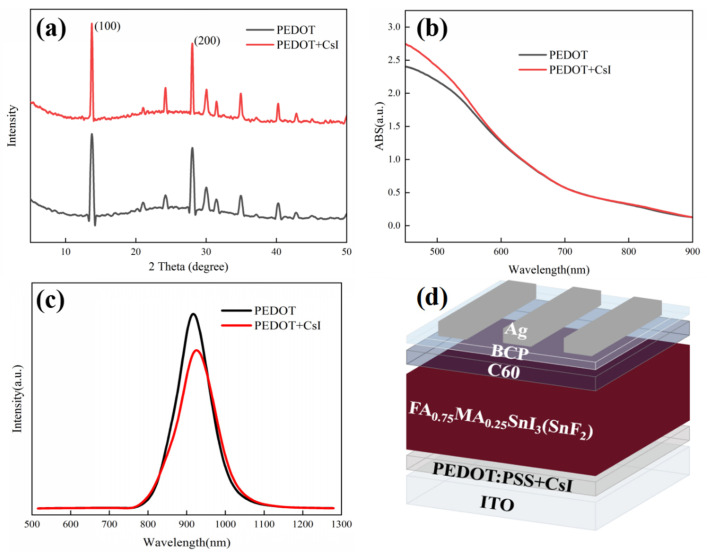
(**a**) XRD spectra of perovskite films deposited on PEDOT and PEDOT + CsI films, (**b**) UV-vis absorption spectra of perovskite films deposited on PEDOT and PEDOT + CsI films, (**c**) PL spectra of perovskite films deposited on PEDOT and PEDOT + CsI films, and (**d**) device configuration of Sn-based PSCs with CsI.

We also investigated the effect of CsI modification on the top perovskite layers deposited on PEDOT:PSS. XRD measurements were employed to evaluate the crystallization of perovskite films deposited on different PEDOT:PSS films in Figure 4a. The position and intensity of diffraction peaks were almost the same for both films, confirming that the doped CsI in the PEDOT:PSS layer has little impact on the bulk crystallization and composition of perovskite film. However, it has been reported that CsI dopant can react with metal halide at the PE-DOT:PSS/perovskite interface and form new perovskite phase. The existing interface phase can passivate the interface defects and facilitate the charge carrier transport [46]. Our experimental results will prove the availability of CsI in modifying PEDOT:PSS film again. In the meantime, UV-vis absorption spectra of perovskite films deposited on different PEDOT:PSS films were also recorded. As shown in Figure 4b, little distinction was observed for the different perovskite films in the whole absorption range. Furthermore, Figure S2 shows the SEM images to distinguish the effect of CsI on the surface morphology of perovskite films. Perovskite films with uniform and compact morphology were observed for both substrates, which is irrelevant to the existence of CsI. The XRD, UV-vis absorption and SEM images of perovskite films confirm that CsI modification does not influence the growth, composition, and the optical property of the perovskite layer deposited on PEDOT:PSS films. However, as mentioned above, the CsI modification is supposed to be beneficial to the interfacial hole extraction and transport. To further elucidate the effect of CsI modification on the charge transfer dynamics, the conductivity of different PEDOT:PSS layers was measured using a structure of ITO/PEDOT:PS/Ag. During the measurements, the thickness of different PEDOT:PSS layers was maintained the same. The J-V results are shown in Figure S3. The PEDOT + CsI film exhibits a higher current density than the PEDOT film under the same bias voltage, indicating a superior conductivity for the PEDOT + CsI film. The improved conductivity of PEDOT + CsI film is favorable to reduce the series resistance of the device, which is helpful for the charge carrier transport throughout the device. PL measurement was further carried out to assess the hole extraction property of different PEDOT:PSS layers with a structure of ITO/PEDOT:PSS/Sn-based perovskite, as shown in Figure 4c. The perovskite film deposited on the PEDOT + CsI layer shows quicker PL quenching than that deposited on the PEDOT layer. It can be inferred that CsI modification can effectively enhance the hole extraction property of the PEDOT:PSS hole-transporting layer. The enhanced hole extraction property of PEDOT + CsI is due to two reasons. The interface perovskite phase resulted from the reaction between CsI and Sn-based perovskite can suppress interface recombination and facilitate hole extraction. And the CsI modification can make the work function of PEDOT: PSS shift toward lower energy, which affords a more favorable bang alignment with the perovskite valence band to facilitate hole extraction [46]. The enhanced hole extraction and transport properties of the PEDOT + CsI layer were supposed to deliver improved photovoltaic performance.

**Figure 5:**
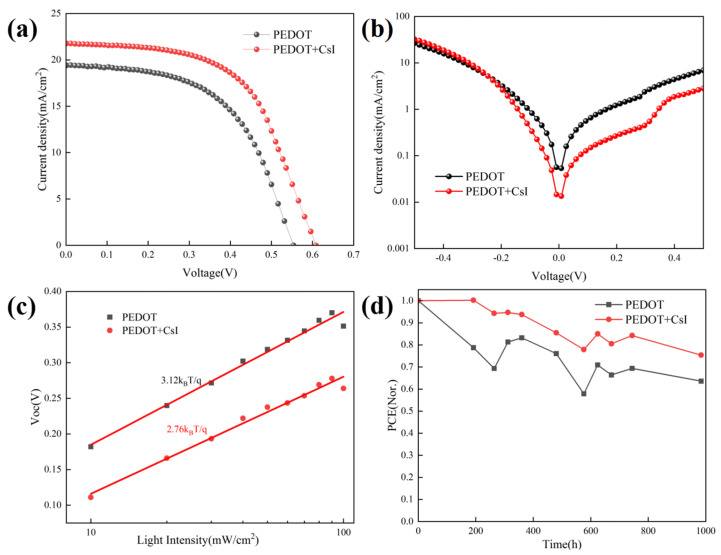
Characterization of device performance with and without CsI: (**a**) J-V curves of the achieved best devices; (**b**) dark J-V measurements; (**c**) Voc vs. illumination intensity; and (**d**) normalized stability test.

The inverted Sn-based PSCs were prepared based on different PEDOT:PSS films to evaluate the effect of CsI modification on device performance. The device configuration of Sn-based PSCs is shown in Figure 4d. Figure 5a presents the J-V curves of the achieved best devices with and without CsI. The device performance based on PEDOT + CsI was obviously improved compared to that based on PEDOT. The detailed photovoltaic parameters are listed in Table 2. The control device based on PEDOT showed a PCE of 5.85%, with a Voc of 553.9 mV, a Jsc of 19.40 mA/cm^2^, and an FF of 54.53%. The optimized PCE of device based on PEDOT + CsI was improved to 7.53%, with a Voc of 608.3 mV, a Jsc of 21.77 mA/cm^2^, and an FF of 56.90%. The improved PCE of device based on PEDOT + CsI was mainly contributed to the increased Voc and Jsc, which is ascribed to the enhanced hole extraction and transport of the PEDOT + CsI layer. Electrochemical impedance spectroscopy (EIS) was performed to further compare the interface charge recombination resistance of devices based on PEDOT and PEDOT + CsI, respectively. Measurements were conducted in the dark and in the frequency range from 10^6^ to 10^−1^ Hz. The obtained Nyquist plots under a bias of 200 mV are provided in Figure S4. The device based on PEDOT + CsI showed a larger arc than the device based on PEDOT, indicating larger recombination resistance and less charge recombination. The EIS results further demonstrated the better interface charge carrier transport and improved photovoltaic performance of the PEDOT + CsI device. To further reveal the enhanced device performance, the dark J-V characteristics were depicted in Figure 5b. The dark current density of the device based on PEDOT + CsI is lower than that of the control device, indicating the effective suppression of device current leakage due to the CsI modification. The measurements of Voc under different illumination intensities were carried out to deepen insights into the interface charge recombination kinetics, as shown in Figure 5c. The ideality factors obtained from the fitted slope were 2.76 and 3.12 for devices with and without CsI, respectively. When the ideality factor is greater than 1, trap-assisted recombination dominates the carrier loss [47]. A lower ideality factor of device with CsI further indicated the effective suppression of interface trap-assisted recombination. All of these results support the improved Jsc, Voc, and FF of the PEDOT + CsI devices. The external quantum efficiency (EQE) was measured to confirm the accuracy of the J-V measurements, as shown in Figure S5. The integrated current densities are 19.20 mA/cm^2^ and 21.38 mA/cm^2^ for the PEDOT and PPEDOT + CsI devices, respectively. The Jsc values obtained from the J-V characteristics were well matched with those obtained by the integration of the spectral response. The stability of Sn-PSCs is still a challenge now. Thus, the device stability in our experiment was also tracked. Firstly, the operation stability for the best-performance PEDOT + CsI device was tracked at the maximum power point (MPP). As shown in Figure S6, the steady-state output current and calculated PCE were 17.40 mA/cm^2^ and 7.32% under 420 mV. Although, the steady-state output PCE was close to the result of J-V measurement, it can be only maintained for 60 s before decay. Therefore, in the follow-up work, it is necessary to further improve the efficiency and stability of Sn-based PSCs. Furthermore, the long-time stability was tracked by storage in a glove box filled with nitrogen for 1000 h without encapsulation. The output PCEs were tested every few days under 1 sun illumination (100 mA/cm^2^), as shown in Figure 5d. The device based on PEDOT + CsI exhibited improved stability while maintaining 75.4% of its initial efficiency compared to 63.6% of the control device. The enhanced stability of PEDOT + CsI device was owed to the improved interface stability between PEDOT + CsI and Sn-based perovskite. The superior hole extraction capability of PEDOT + CsI can facilitate charge carrier transport and reduce carrier accumulation at the interface, which was favorable to slow down the interface degradation, delivering enhanced long-time stability of device. 

## 3. Materials and Methods

### 3.1. Materials

Formamidine iodide (FAI, 99.5% purity), methylammonium iodide (MAI, 99.5% purity), cesium iodide (CsI, purity 99.999%), and tin iodide (SnI_2_, purity 99.999%) were purchased from China Yingkou Advanced Materials Technology Co., Ltd. (Yingkou, China). Indium tin oxide (ITO, film resistance: 7 ohms/square), alkali root alkali (BCP), and C60 were purchased from Xi’an Yoli Solar Energy Co., Ltd. (Xi’an, China). Tin fluoride (SnF_2_, purity 99.9%) was purchased from Shanghai McLean Biochemical Technology Co., Ltd. (Shanghai, China). PEDOT:PSS 4083 (1.3–1.7 wt %), chlorobenol (CBZ, anhydrous, 99.8% purity), dimethyl sulfoxide (DMSO, anhydrous, 99.9% purity), and N, N-dimethylformamide (DMF, anhydrous, 99.8% purity) were purchased from Beijing J&K scientific LLC (Beijing, China). Silver (Ag, purity 99.99%) was procured from Beijing New Metal Materials Technology Co., Ltd. (Beijing, China). All purchased materials are used directly without further purification.

### 3.2. Device Fabrication

Perovskite precursor: 0.8 M/L FA_0.75_MA_0.25_SnI_3_ perovskite precursor solution was prepared with mixed FAI (103.18 mg), MAI (31.79 mg), SnI_2_ (298 mg,), and SnF_2_ (0 M, 0.02 M, 0.03 M and 0.04 M) in a mixture of 1 mL anhydrous DMF and anhydrous DMSO (DMF:DMSO = 4:1). FA_0.75_MA_0.25_SnI_3_ perovskite precursor solution was stirred overnight in a shaker at room temperature and filtered with 0.22 μm polytetrafluoroethylene (PTFE) filter before use. All preparation processes were carried out in nitrogen-filled glove boxes (O_2_ < 0.1 ppm, H_2_O < 0.1 ppm).

Device fabrication: First, the ITO glass sheet was cleaned with a professional dishwashing liquid and then subjected to ultrasound with deionized water, ethanol, and isopropyl alcohol for 15 min. After cleaning, the glass was dried with nitrogen gas, and then, ITO was irradiated with UV for 20 min. To prepare the hole-transporting layer, we first filtered PEDOT:PSS (with and without CsI) using a 0.45 µm polyether sulphone (PES) filter, and then, it was spin-coated onto the ITO surface at 4000 rpm for 30 s and annealed at 150 °C in ambient air for 30 min. Then, ITO with prepared hole-transporting layer was transferred into the glove box to prepare the perovskite layer. A 40 µL volume of the perovskite precursor solution was rotated on the surface of PEDOT:PSS at 5000 rpm for 60 s. A 120 µL volume of chlorobenzene (CBZ) was rapidly dripped onto the substrate starting at 15 s and then annealed at 90 °C for 15 min. C60 (35 nm) and BCP (8 nm) were prepared by thermal evaporation under high vacuum at 10^−4^ Pa. Finally, an 80 nm thick Ag electrode was thermally evaporated as a cathode under a high vacuum of 10^−4^ Pa. The active area of perovskite solar cells is 5 mm^2^.

## 4. Conclusions

In summary, crystallization and interface engineering were used to synergistically modulate the performance of Sn-based perovskite solar cells with an inverted configuration of ITO/PEDOT:PSS/FA_0.75_MA_0.25_SnI_3_/C60/BCP/Ag. When optimized 0.03 M SnF_2_ was added to the perovskite precursor solution, the crystallization of Sn-based perovskite film was obviously improved with uniform morphology and large grains. At the same time, CsI was applied to modify the PEDOT:PSS hole-transporting layer. The CsI modification can enhance the hole extraction and transport of PEDOT:PSS, which can optimize the interface contact and inhibit the interface recombination. In the synergistic effect of SnF_2_ additive and CsI modification, the inverted Sn-based PSCs show a power conversion efficiency of 7.53% with enhanced device stability.

## Figures and Tables

**Table 1:** Photovoltaic parameters of Sn-based PSCs based on different SnF_2_ concentrations.

SnF_2_	Voc (mV)	Jsc (mA/cm^2^)	FF (%)	PCE (%)
0 M	204.7	16.15	48.45	1.60
0.02 M	346.2	17.88	37.23	2.30
0.03 M	439.6	19.04	55.93	4.68
0.04 M	382.3	16.77	40.02	2.57

**Table 2:** Photovoltaic parameters of the achieved best devices with and without CsI.

Device	Voc (mV)	Jsc (mA/cm^2^)	FF (%)	PCE (%)	Average PCE (%)
PEDOT	553.9	19.40	54.53	5.86	5.13%
PEDOT + CsI	608.3	21.77	56.90	7.53	6.85%

## Data Availability

The data that support the findings of this study are available from the corresponding authors upon reasonable request.

## Supplementary Materials

The following supporting information can be downloaded at: https://www.mdpi.com/article/10.3390/molecules29112557/s1, Figure S1: Statistical distribution of PCE for Sn-PSCs based on PEDOT:PSS with different CsI concentrations; Figure S2: (a,b) SEM images of perovskite films deposited on PEDOT film at 10 KX and 20 KX. (c,d) SEM images of perovskite films deposited on PEDOT + CsI film; Figure S3: Hole transport property measurement for different PEDOT:PSS films; Figure S4: Nyquist plots at 200 mV for Sn-based PSCs based on PEDOT and PEDOT + CsI; Figure S5: EQE spectra and integrated current density of the best-performance Sn-based PSCs based on PEDOT and PEDOT + CsI; Figure S6: The steady-state output current and calculated PCE of the best-performing PEDOT + CsI device at the maximum power point (MPP).
