# Supplementary material for: Synergistic Modulation of Sn-Based Perovskite Solar Cells with Crystallization and Interface Engineering
Source: Molecules. 2024 May 29;29(11):2557. doi: 10.3390/molecules29112557 (PMC11173692; doi:10.3390/molecules29112557)
Supplement: Supplementary file 1 [file molecules-29-02557-s001.zip › molecules-2993330-supplementary.pdf]

Supporting Information

Synergistic Modulation of Sn-Based Perovskite Solar Cells with Crystallization and Interface Engineering

Yunzhao Sun, Yaoyao Song, Mengfan Liu, Huiyin Zhang *

School of Instrument Science and Opto-Electronics Engineering, Beijing Information Science &Technology University, Beijing 100192, China

*Corresponding author:

E-mail address: zhy@bistu.edu.cn

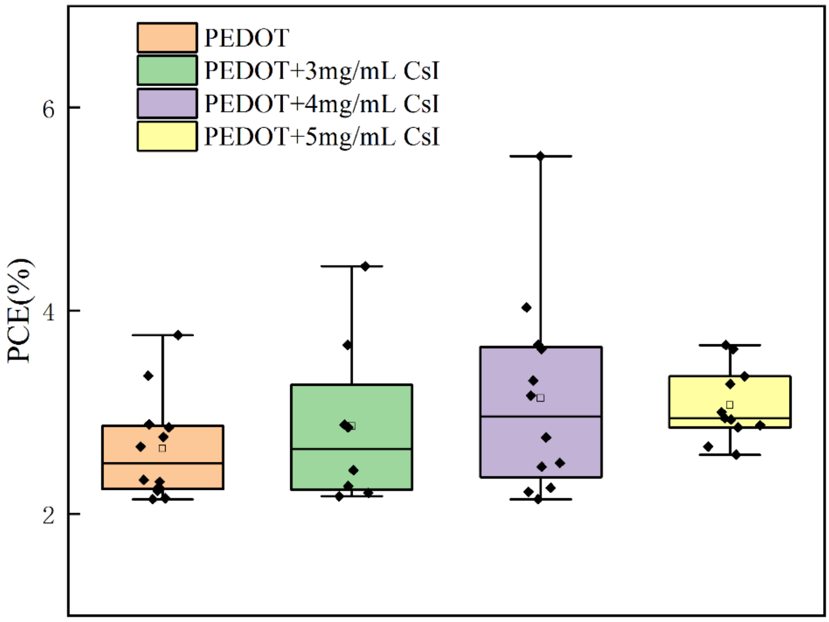

Figure S1. Statistical distribution of PCE for Sn-PSCs based on PEDOT:PSS with different CsI concentrations.

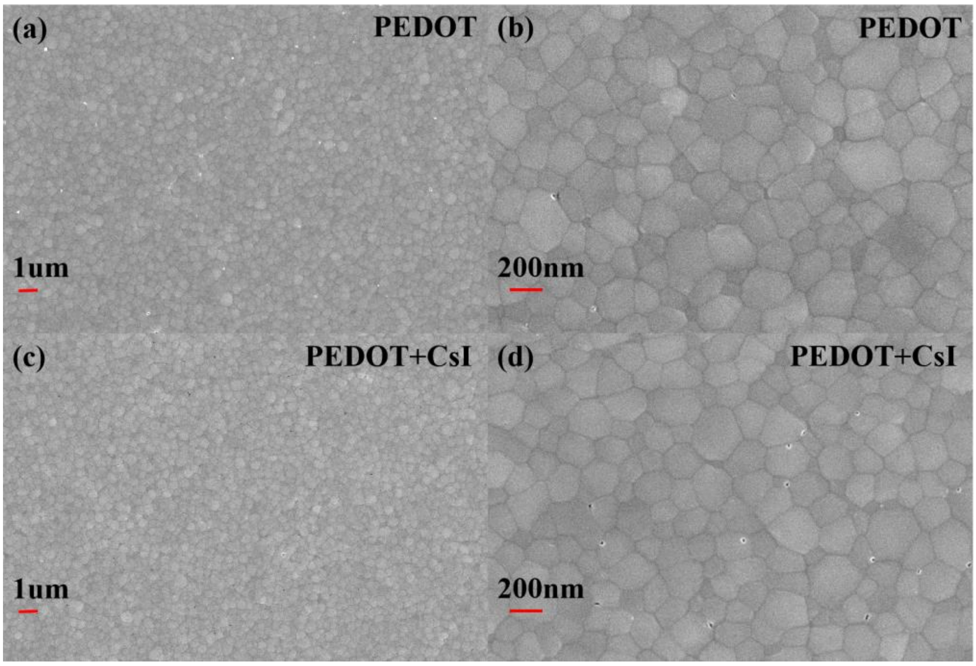

Figure S2. (a) and (b) SEM images of perovskite films deposited on PEDOT film at 10KX and 20KX. (c) and (d) SEM images of perovskite films deposited on PEDOT+CsI film.

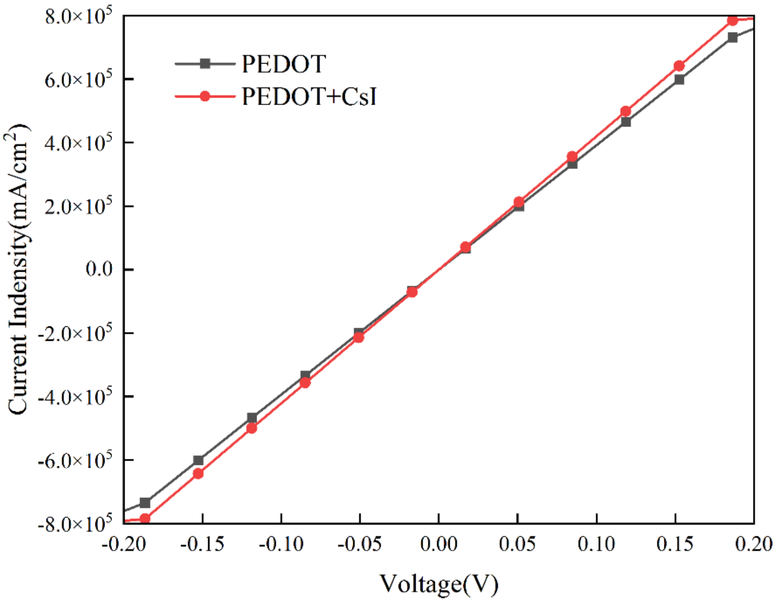

Figure S3. Hole transport property measurement for different PEDOT:PSS films.

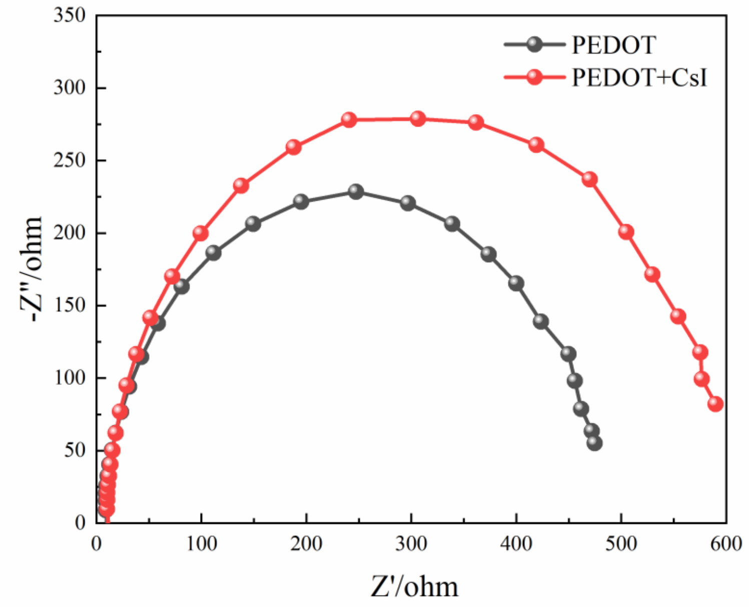

Figure S4. Nyquist plots at 200mV for Sn-based PSCs based on PEDOT and PEDOT+CsI.

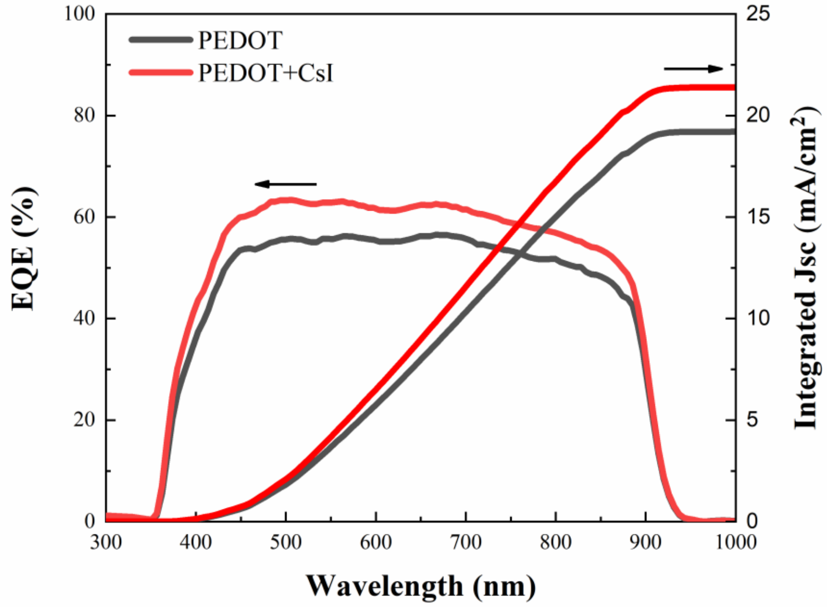

Figure S5. EQE spectra and integrated current density of the best-performance Sn-based PSCs based on PEDOT and PEDOT+CsI.

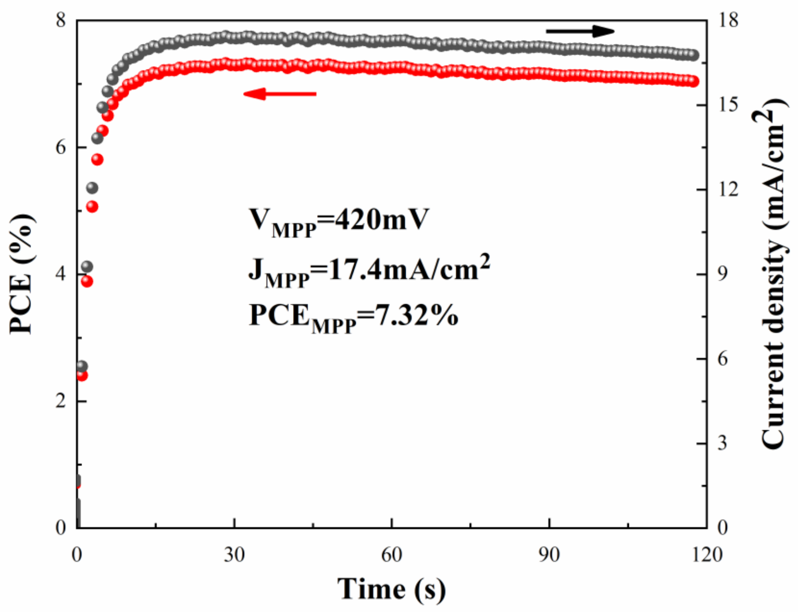

Figure S6. The steady-state output current and calculated PCE of the best-performing PEDOT+CsI device at the maximum power point (MPP).
